# A resonant valence bond spin liquid in the dilute limit of doped frustrated Mott insulators

**DOI:** 10.1038/s41567-025-02923-8

**Published:** 2025-05-29

**Authors:** Cecilie Glittum, Antonio Štrkalj, Dharmalingam Prabhakaran, Paul A. Goddard, Cristian D. Batista, Claudio Castelnovo

**Affiliations:** 1 T.C.M. Group, Cavendish Laboratory, University of Cambridge, Cambridge, UK; 2 Department of Physics, Faculty of Science, University of Zagreb, Zagreb, Croatia; 3 Department of Physics, University of Oxford, Oxford, UK; 4 Department of Physics, University of Warwick, Coventry, UK; 5 Department of Physics and Astronomy, University of Tennessee, Knoxville, TN USA; 6 Neutron Scattering Division, Oak Ridge National Laboratory, Oak Ridge, TN USA

**Keywords:** Electronic properties and materials, Topological defects, Theoretical physics

## Abstract

Ideas about resonant valence bond liquids and spin–charge separation have led to key concepts in physics such as quantum spin liquids, emergent gauge symmetries, topological order and fractionalization. Despite extensive efforts to demonstrate the existence of a resonant valence bond phase in the Hubbard model that originally motivated the concept, a definitive realization has yet to be achieved. Here we present a solution to this long-standing problem by uncovering a resonant valence bond phase exhibiting spin–charge separation in realistic Hamiltonians. We show analytically that this ground state emerges in the dilute-doping limit of a half-filled Mott insulator on corner-sharing tetrahedral lattices with frustrated hopping, in the absence of exchange interactions. We confirm numerically that the results extend to finite exchange interactions, finite-sized systems and finite dopant density. Although much attention has been devoted to the emergence of unconventional states from geometrically frustrated interactions, our work demonstrates that kinetic energy frustration in doped Mott insulators may be essential for stabilizing robust, topologically ordered states in real materials.

## Abstract (web summary)

The concept of resonant valence bond phases has inspired many areas of condensed matter physics, but few realistic models have been identified. Now an analytical solution of such a phase has been found for pyrochlore and related lattices.

## Main

Inspired by Pauling’s early theory of resonant covalent-bond-sharing in aromatic molecules, such as benzene, and by Bethe’s antiferromagnetic linear chain, Fazekas and Anderson^1,2^ proposed in the early 1970s a possible competitor to Néel order in antiferromagnetically coupled spins: a state where spins are valence-bond paired into singlets that resonate quantum mechanically among different pairing configurations, thus compensating for the partial loss of antiferromagnetic energy. The result is a resonant valence bond (RVB) state. This exciting idea gained further momentum with the discovery of high-temperature superconductivity about a decade later and with the prompt proposal that it may indeed be underpinned by spin–charge separation^3–8^ in a system of strongly correlated electrons in an RVB liquid phase (for a review, see refs. ^9,10^). The appeal was so great that for decades to follow, numerous physicists have been looking for evidence of this suggestion ‘along a bewildering variety of routes’^9^.

Aside from its relevance to high-temperature superconductivity, the quest for an RVB liquid state has led to several fundamental advances, many of which play a central role in our understanding of modern physics. Of particular import, the notion of spin liquids and its relation to deconfinement in gauge theory have been greatly clarified^10,11^ and subsequently extended to further areas of research^12–15^, thus encompassing new concepts such as topological order and fractionalization^16^.

Intense work has continued throughout the years, yet Anderson’s original proposal has hitherto evaded discovery. Following the seminal work by Moessner and Sondhi^12^, which established the possibility of a short-range liquid phase in quantum dimer models^17^, several efforts have been made to translate this result into a system of SU(2) spins by building on Klein models^18^ and effective and decorated dimer models^19–22^. It is believed that magnetic frustration in quasi-two-dimensional (quasi-2D) Mott insulators could be responsible for RVB physics; however, parent Hamiltonians that host an exact RVB ground state are often unphysical (see, for example, ref. ^23^).

In this work, we found a concise and elegant solution that unintentionally fulfils the inspirational words of ref. ^20^: ‘A more important task perhaps, now that the question of principle is settled, is to refocus on studying much simpler Hamiltonians.’ We demonstrate that an RVB liquid phase, which exhibits spin–charge separation, emerges naturally upon dilute-doping a large-*U* (on-site Coulomb repulsion) Hubbard model on a lattice of corner-sharing tetrahedra—whether a planar chequerboard or a three-dimensional pyrochlore lattice—near half-filling. This result hinges on kinematic spin correlations set by frustrated hopping and is an instance of the so-called counter-Nagaoka effect^24^. Without loss of generality, in the following we shall consider the case of hole doping; the same can be said for electron doping when the hopping amplitude has the opposite sign. We proved our claims by finding an exact lower bound to the Hamiltonian energy and by explicitly constructing an RVB liquid state that precisely matched it in the thermodynamic single-hole limit. We confirmed this behaviour by numerically investigating systems of finite size. (A numerical study of one- and two-hole doping in the *t*–*J* model on the chequerboard lattice had already been carried out by Poilblanc^25^, where *t* is the electron hopping amplitude and *J* the nearest-neighbour exchange interaction strength. Poilblanc’s work highlighted the −4*t* and −8*t* respective ground-state energies and described how the singlet background somehow changes the effective hopping, which is a precursor to gaining the understanding in terms of RVB liquid and two *π*-flux attachment presented in this work.) Here we propose a way to construct single-hole and two-hole RVB-liquid ground-state wavefunctions for any system size. These seem to be exact within numerical accuracy, a truly notable feat for a strongly correlated electron problem. Finally, we show numerically that our results are stable in systems of finite hole density as well as in the presence of sufficiently small exchange interactions between the spins.

The crucial role of kinetic energy frustration in stabilizing a topologically ordered RVB liquid state is a notable outcome of our findings, which provide a hitherto-unexplored way to connect dimer coverings with solutions to the Hubbard model. Our results have the potential to shift the traditional paradigm, which has predominantly considered exchange frustration as the mechanism for stabilizing quantum spin liquids (QSLs). In other words, doping insulators that lie deep within the Mott regime with a single type of carrier (electrons or holes, depending on the sign of the hopping amplitude) promises to become a guiding principle for the discovery of spin liquid states in correlated matter.

Our Hamiltonian is eminently simple and realistic, and it is of direct relevance to several experimental settings as well as synthetic quantum platforms^26–28^. We identify in particular some families of pyrochlore compounds as suitable frameworks for experimentally testing our predictions and suggesting how it may be possible to manifest the elusive RVB state in these materials.

## Results

**Fig. 1:**
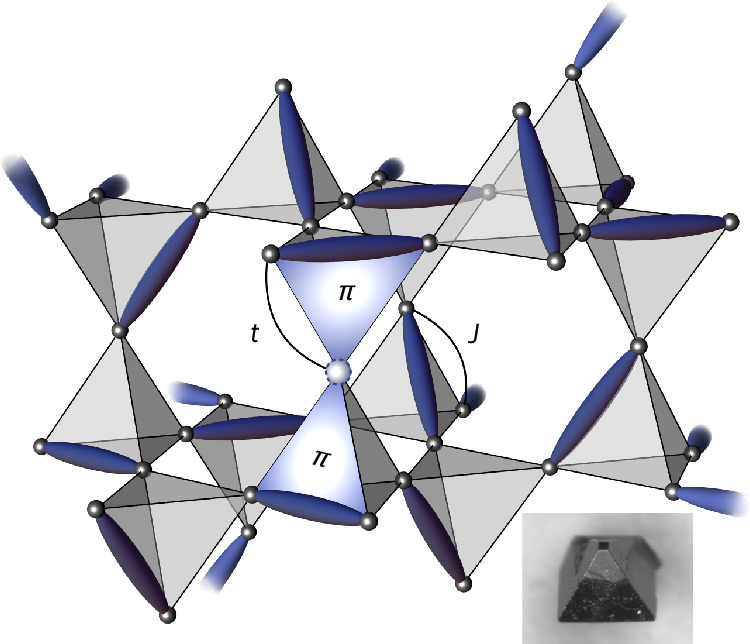
Pictorial illustration of one of the dimer-singlet states. These states participate in the superposition forming the RVB liquid ground state of the *t*–*J* model, equation (1), on a lattice of corner-sharing tetrahedra. Singlets are indicated by dark thin ellipsoids. A holon at a given location is indicated by a light sphere. The *π*-fluxes depict the phase induced by the singlet coverings (relative to a ferromagnetic background) when the holon moves around the corresponding triangular plaquette. Inset, an Y_2_Ir_2_O_7_ pyrochlore crystal grown by D. Prabhakaran in Oxford.

We study the *t*–*J* model on a lattice of corner-sharing tetrahedra (Fig. 1) near half-filling, with Hamiltonian

1 $$\hat{{\mathcal{H}}}=-t\sum _{\left\langle i,\,j\right\rangle \sigma }\left[{\hat{c}}_{i\sigma }^{\dagger }{\hat{c}}_{j\sigma }+\mathrm{h.c.}\right]+J\sum _{\left\langle i,\,j\right\rangle }{\hat{{\bf{S}}}}_{i}\cdot {\hat{{\bf{S}}}}_{j},$$

where 〈*i*, *j*〉 denotes nearest-neighbour pairs of sites; ${\hat{c}}_{i}^{\dagger }$ (${\hat{c}}_{i}$) is the constrained fermionic creation (annihilation) operator at site *i* acting in the space where double occupancy is strictly forbidden (Supplementary Information Note C). ${\hat{{\bf{S}}}}_{i}$ is the corresponding spin operator, ${\hat{{\bf{S}}}}_{i}=\frac{1}{2}{\sum }_{\sigma ,{\sigma }^{{\prime} }}{\hat{c}}_{i\sigma }^{\dagger }{{{\mathbf{\upsigma}}}}_{\sigma \sigma^{\prime} }{\hat{c}}_{i\sigma^{\prime} }$. Here *σ* labels the states of spin-1/2 degrees of freedom, and **σ** is the conventional vector of Pauli matrices. For generality, we treat the hopping *t* and the spin exchange *J* as independent parameters. Note that for fermions and our convention in equation (1), hole doping with *t* > 0 or electron doping with *t* < 0 correspond to frustrated hopping when the elementary loop of the lattice has an odd number of sites. In the following we shall focus, without loss of generality, on hole doping and *t* > 0.

Our results apply straightforwardly, for example, to the 2D case of the chequerboard lattice or to the three-dimensional case of the pyrochlore lattice. Note that for the 16-site system considered below, the two lattices, in fact, coincide.

Our first result is analytical, derived in the thermodynamic limit for a single hole and *J* = 0. As demonstrated in detail in Supplementary Information Note A, the spectrum of the Hamiltonian in equation (1) has a strict lower bound of −4*t*. Furthermore, we prove that this bound is met by the RVB spin liquid ground state:

2 $$\left\vert {\varPsi }_{{\rm{RVB}}}\right\rangle \propto \sum_{{\bf{r}}\{{a}_{{\bf{r}}}\}}\prod_{\left\langle i,\,j\right\rangle \in {a}_{{\bf{r}}}}{\hat{d}}_{ij}^{\dagger }\left\vert 0\right\rangle,$$

where **r** spans all possible positions of the holon; *a*_**r**_ is a generic dimer covering of the lattice with a holon at **r** subject to the condition that there is one and only one dimer per tetrahedron (see, for example, Fig. 1); and ${\hat{d}}_{ij}^{\dagger }=\frac{1}{\sqrt{2}}({\hat{c}}_{i\uparrow }^{\dagger }{\hat{c}}_{j\downarrow }^{\dagger }-{\hat{c}}_{i\downarrow }^{\dagger }{\hat{c}}_{j\uparrow }^{\dagger })$. We shall refer to each state in the superposition forming |$\varPsi$_RVB_〉 as a dimer-singlet state. The kinetic energy frustration disappears for the opposite sign of the hopping amplitude, which is equivalent to inserting a *π*-flux on each triangular plaquette of the pyrochlore lattice. The RVB wavefunction minimizes the holon kinetic energy by spontaneously generating a *π*-flux on the triangular face containing the hole and a singlet. Namely, the RVB wavefunction binds two *π*-fluxes (fluctuating with the dimer-singlet resonant states) to each hole to lower its kinetic energy to −4*t* by partially lifting the frustration (Fig. 1)^25^.

This is an instance of the so-called counter-Nagaoka effect^24^, whereby frustrated quantum hole hopping leads to antiferromagnetic correlations in a Mott insulator near half-filling. The phenomenon has been shown to generically lead to conventionally ordered phases in two dimensions^29–32^, with the notable exception of a Husimi cactus of triangles (akin locally to a kagome lattice), where Kim proved that it leads to a regular valence bond pattern in the presence of a single (deconfined) hole^33^. It has not been thus far investigated in three dimensions, and it is all the more notable to see it favour a QSL state in lattices of corner-sharing tetrahedra (for a brief review of the counter-Nagaoka effect, see Supplementary Information Note B).

Note that, unlike the triangular case considered in ref. ^33^, where the dimer-singlet configuration is fully determined once the hole position is fixed, for our corner-sharing tetrahedra, there are extensively many dimer singlets forming a massively entangled superposition of electronic states for each fixed hole position. The latter is exactly what one expects of an RVB liquid state.

The ground state of equation (2) is exact in the thermodynamic limit, where such dimer coverings with a single monomer are well defined. In a finite lattice of corner-sharing tetrahedra with periodic boundary conditions, introducing a hole necessarily requires an unpaired spin. As expected for an RVB ground state, the spin and the charge of the fermion added to the Mott insulator separate. That is, the probability of finding the holon within a finite distance from the spinon is zero in the thermodynamic limit. The spinon must necessarily occupy a passive site of the tetrahedron, implying that the holon will never visit the site occupied by the unpaired spin. In other words, the spinon is expected to have infinite mass (static spinon), and the spinon–holon interaction is expected to be repulsive. Both observations are supported by our numerical analysis, which gives our second set of results and attests the stability of our RVB liquid phase in finite-sized systems, finite hole densities and in the presence of interactions.

We studied the behaviour of equation (1) in finite pyrochlore systems using exact diagonalization on a 16-site cubic cell and using a density matrix renormalization group (DMRG)^34–36^ on a 32-site system with periodic boundary conditions (for details of the study, see Supplementary Information Note D). Notably, we found that the ground-state energy bound −4*t* was met (when *J* = 0) for all system sizes, within numerical precision. We observed a non-trivial degeneracy (18 states for 16 sites and 34 states for 32 sites). Consistent with the analytical considerations above, we found a unique ground state (again with energy −4*t*) when the unpaired spin was pinned to a given lattice site.

Within numerical accuracy, we show that the ground-state wavefunction with a fixed spin is given by the equal-amplitude superposition of all dimer-singlet configurations (constrained to no more than one dimer per tetrahedron) with a delocalized holon, for our 16-site and 32-site systems (Supplementary Information Note D). This allowed us to propose an exact wavefunction for the single-hole case for all system sizes, which is a highly unusual feat. Such a state has a vanishing expectation value of the projector onto the maximum angular momentum for each tetrahedron with no hole^19,20^ (see also Kivelson, S. A., private communication, unpublished), which we verified (Supplementary Information Note D).

**Fig. 2:**
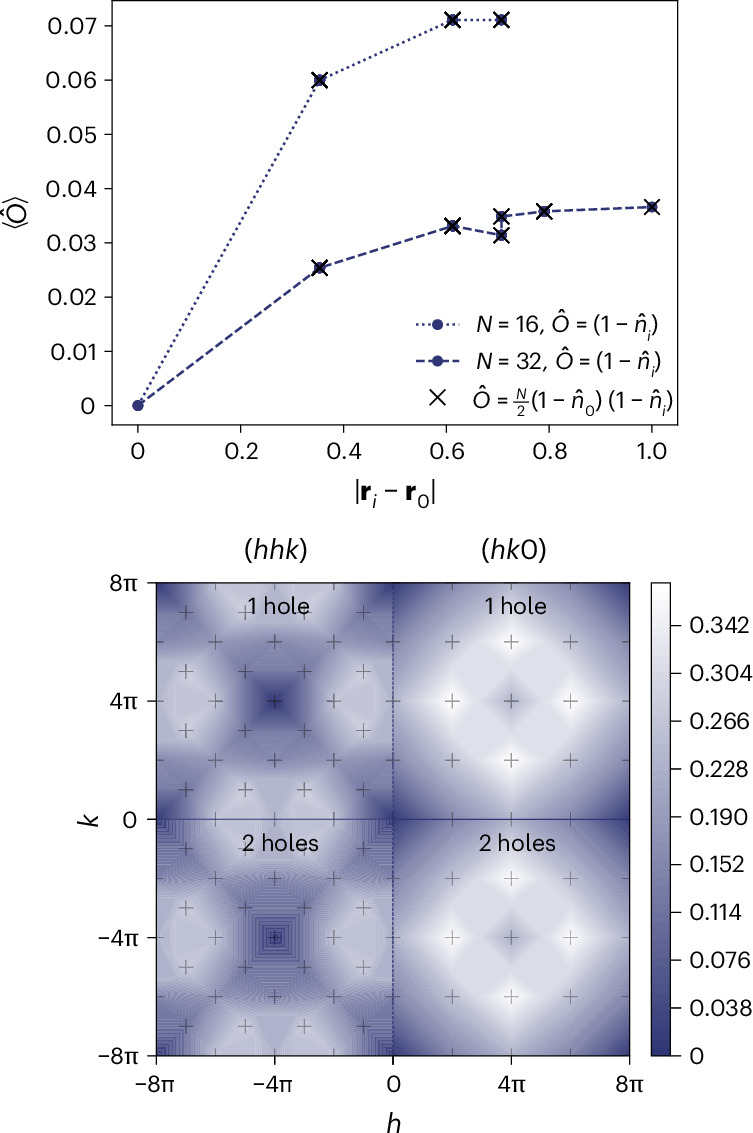
Correlations and spin structure factor in the RVB state. Top, blue dots show the holon density $\langle{\hat{O}}\rangle = \langle 1-{\hat{n}}_i \rangle$ ($\hat{n}_i$ being the electron density operator) for a 16-site and a 32-site pyrochlore system doped with a single hole as a function of distance from the pinned spin, $\vert {\bf{r}}_{i} - {\bf{r}}_{0} \vert$, for system sizes $N=16, \, 32$. It indicates that the spinon and holon separate (the two different values at distance ~0.7 are due to the two inequivalent third-nearest-neighbour sites on the pyrochlore lattice). Crosses show the holon–holon correlations when there are two holes present in the system. They are numerically identical to the holon–spinon correlations. Bottom, spin structure factor in the (*hhk*) and (*hk*0) reciprocal lattice planes for the 32-site pyrochlore system with one hole (top half) and two holes (bottom half) for *t* = 1 and *J* = 0. Accessible momenta are marked by grey pluses.

As illustrated in the top panel of Fig. 2, the holon density shows a weak repulsive dependence on the distance from the spinon in finite-sized systems, in agreement with spin–charge separation in the thermodynamic limit as argued earlier on analytical grounds (Supplementary Information Note D). In the bottom panel of Fig. 2, we show the spin structure factor of our numerical ground states. Characteristic differences that could be measured experimentally seemingly distinguish it from, for example, the classical or quantum Heisenberg spin liquid structure factors (Supplementary Information Note D); however, future work on larger systems is needed if we are to make more precise statements about this. On general grounds, we expect exponentially decaying spin correlations; the nature of the singlet correlations is less clear, as the correspondence with spin ice states^20,37–39^ could induce power-law behaviour and an emergent U(1) gauge symmetry with gapless photon excitations. This is, however, far from trivial due to the non-orthogonality of the dimer-singlet states.

We briefly studied the case *J* = 0 and two holes, which we were able to access numerically in the 16-site and 32-site systems. We observed no tendency towards holon attraction (see top panel of Fig. 2 and Supplementary Information Note E). The ground state was then unique, with energy −8*t*, indicating that the second holon was able to occupy the site of the unpaired spin and to render it itinerant, irrespective of the presence of the other holon. Consistently, we found that the holon–spinon correlations for a single hole were numerically identical to the holon–holon correlations for two holes (Fig. 2). The nature of the two-hole ground state can be understood from the perspective of the *π*-flux binding discussed earlier. A holon can move only on the tetrahedron around the triangular face that contains a singlet (Fig. 1), and it is forbidden by interference effects to visit the fourth tetrahedral site (thus rendering two holons on the same tetrahedron blind to one another). In other words, the single-hole RVB wavefunction is an equal weight superposition of the holon propagating in all possible sublattices that can be constructed by choosing one triangle of each tetrahedron that is visited. For the two-hole RVB wavefunction (namely, the equal-amplitude superposition of all dimer-singlet states hosting two holons, with no more than one dimer per tetrahedron), the holons propagate through different triangles when they meet at the same tetrahedron, meaning that the effective coordination number is still equal to 4 for each of them and that the energy is 2 × (−4*t*) = −8*t*. These observations, in particular the coincidence of the spinon–holon and holon–holon correlators in Fig. 2, strongly indicate that the holon excitations in our RVB state are bosonic.

We verified numerically that the proposed RVB wavefunction is also the exact ground state for the two-hole system (for 16 sites and 32 sites). The argument does not extend to three or more holes on finite systems, and their behaviour is left to future investigations.

Intriguingly, the opposite (stoichiometric) limit of vanishing hole density is singular for the 16-site and 32-site systems in question. The wavefunction given by the equal-amplitude superposition of all dimer-singlet states with one and only one dimer per tetrahedron vanishes identically (Supplementary Information Note A). Note that the vanishing also occurs for equal-amplitude superpositions of all dimer-singlet states, without the constraint of one dimer per tetrahedron. The RVB liquid phase, therefore, seems to exist solely in the presence of hole doping (at least within the remit of our accessible system sizes and periodic boundary conditions), and it can be stabilized only by kinetic energy frustration.

**Fig. 3:**
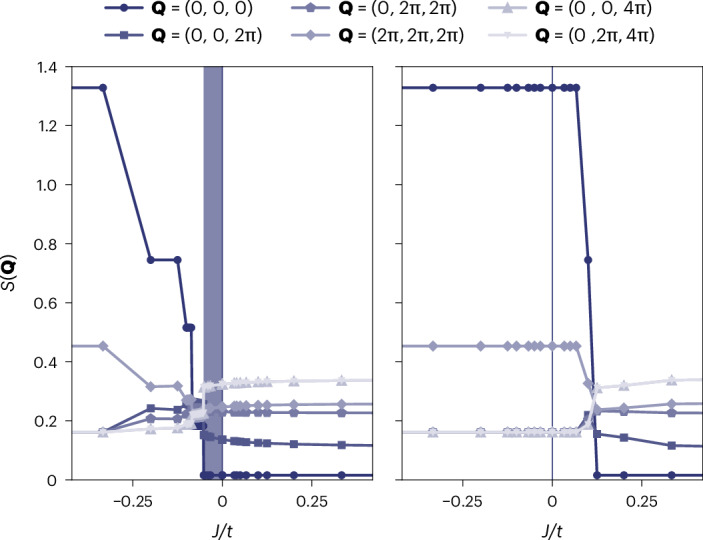
Spin structure factor. Dependence of the ground-state spin structure factor *S*(**Q**) (for all individual reciprocal lattice points **Q**) on *J*/*t* (*t* > 0) for a 16-site system. Left, the system is doped with one hole. The region where the correlations are antiferromagnetic, despite the ferromagnetic *J*, is shaded. Right, the system is doped with one electron, for comparison.

Finally, we looked at the concomitant effects of *t* and *J* in the system. We considered a 16-site system with a single hole. We fixed *t* = 1 and varied *J*, spanning both positive and negative values. In Fig. 3, we plot the behaviour of the ground-state spin structure factor for all distinct points in reciprocal space. We found that the spin-liquid behaviour persists down to a finite negative value of (*J*/*t*)_c_ ≈ −0.052 (for holon density 6.25%), indicating that a transition from ferromagnet to QSL can be induced on the pyrochlore lattice by frustrated hole doping. It has been argued that the effective doping-induced antiferromagnetic interaction between the spins is proportional to the holon density at sufficiently small concentrations^24^, and we expect our results to correspondingly become more pronounced (with the caveat of possible phase separation effects as in ref. ^40^ for the large but finite *U* Hubbard limit). Indeed, on adding a second hole to the 16-site system, we found (*J*/*t*)_c_ ≈ −0.173 (holon density 12.5%; Supplementary Information Note E). Figure 3 compares for reference the case of electron doping, which is not frustrated for *t* > 0 and exhibits a kinetic tendency towards ferromagnetic order through the Nagaoka effect.

## Discussion

Our results provide a simple and elegant solution to a problem that has eluded researchers for nearly half a century. Through exact results and detailed numerical simulations, we demonstrated that the long-sought-after RVB spin liquid state exhibiting spin–charge separation emerges naturally as a ground state of a large-*U* Hubbard Hamiltonian on lattices of corner-sharing tetrahedra upon dilute-doping away from the Mott insulating state. The key aspect of this emergent behaviour is the kinetic energy frustration of carriers that result from doping the Mott state.

The non-Fermi-liquid behaviour (for example, spin–charge separation) of such an RVB liquid state implies that the large-*U* limit of the pyrochlore Hubbard model cannot be smoothly connected to its weak-coupling counterpart (small *U*). Hence, an effective description at large *U*, which is non-perturbative in nature, is essential for fully characterizing it. It is interesting to contrast it to the Fermi-liquid behaviour argued to occur in spin liquid states stabilized by frustration in lattices of corner-sharing tetrahedra near half-filling in finite-*U* Hubbard models (see, for example, refs. ^41,42^). This raises the intriguing possibility of a crossover between the two regimes as a function of doping.

The eminently neat and realistic nature of the Hamiltonian opens the door to further theoretical investigations into the physics of RVB phases, possibly also with the help of implementations on synthetic quantum platforms^27,28^ (note in particular how the leading RVB-liquid behaviour is already apparent in relatively small systems of 16 sites). The rare availability of an exact single-hole and two-hole ground-state wavefunction for systems of any size provides an uncommon capability for comparing theory and simulation experiments.

It should also be possible to test our results in real materials. In the first instance, an experimental comparison between the effects of hole and electron doping, that is, switching between counter-Nagaoka antiferromagnetic correlations and Nagaoka ferromagnetic ones^43^ (as shown in Fig. 3), could be used to check if a given Mott insulator is indeed in the large-*U* regime relevant to this work. The dynamical spin structure factor *S*(**Q**, *ω*) of the predicted RVB state could be experimentally accessed through neutron scattering, as a function of reciprocal lattice vector **Q** and angular frequency *ω*. The low-energy region, in particular, ought to include a spinon continuum, flat in the infinite-*U* limit and broadened by *J*, which could be used to measure spin interactions. The spin–spin correlation length could be directly extracted from the static spin structure factor *S*(**Q**) = ∫ d*ω* *S*(**Q**, *ω*). The spin–charge separation could be indirectly measured with photoemission experiments, which would reveal a vanishing weight of the quasiparticle peak and could also be used to extract the hopping amplitude *t*. Finally, in the presence of interactions (for example, exchange *J*), the undoped Mott insulating phase will probably exhibit magnetic properties that are notably different from those of the RVB liquid phase. A transition between the two phases as a function of doping could be detected from signatures in thermodynamic properties, such as specific heat and susceptibility, as well as in neutron scattering or muon-spin rotation measurements, which raises the tantalizing prospect of observing hole-induced QSL behaviour. If the interactions are ferromagnetic, one could envisage looking for polaronic behaviour and possibly magnonic Cooper pairs (bound states of two fermions and one magnon) in the ferromagnetic phase close to the transition to the RVB state^31,44^.

Candidate materials that realize large-*U*-Hubbard-model Mott insulators on lattices of corner-sharing tetrahedra are not commonplace. One can, nonetheless, identify families of compounds, which, although they do not immediately map onto our model, provide promising avenues for further investigation and the possible realization of the RVB-liquid behaviour presented in our work. One such family of compounds, the 5*d* transition-metal pyrochlore oxides, has been extensively studied and is known to form regular pyrochlore lattices. Rare-earth iridates and ruthenates, RE_2_TM_2_O_7_, where RE is a rare earth and TM a transition metal, host 5*d*^5^ TM^4+^ ions with an effective angular momentum *J*_eff_ = 1/2 ground-state doublet. Holons can be introduced by doping on the non-magnetic RE site, thus effectively replacing TM^4+^ with TM^5+^, *J*_eff_ = 0 ions. Some doping experiments have already been performed with these materials, including measurements on polycrystalline Y_2−*x*_Ca_*x*_Ir_2_O_7_ for *x* = 0–0.2 (refs. ^45,46^), for which a marked effect on the magnetic and transport properties was observed. However, the polycrystalline nature of the samples used in those studies seems to have led to confusing and contradictory results (see Supplementary Information Note G for more details). Recent progress in producing high-quality single crystals of this material family (see, for example, refs. ^47–49^ and inset of Fig. 1) could provide opportunities to understand these changes and perhaps observe evidence for the predictions discussed above. (See Supplementary Information Note G for a more detailed review of the materials that could potentially be investigated, the methods by which they could be grown and possible experimental routes to measure a doping-induced order-to-QSL transition.) Having said this, certain caveats should be flagged regarding the applicability of our theoretical results to these 5*d* compounds: they tend to be weak insulators, and they display strong spin–orbit effects. Hence, they are less likely to be described well by models of isotropic hopping. Further theoretical work is needed to study the tolerance of the original model to these departures. Another family of compounds involves 3*d* transition metals, which offer large *U*/*t* ratios and (owing to weak spin–orbit coupling) near-isotropic hopping intrinsic to our model. Investigating pyrochlore lattices of spin-1/2 Cu(ii) is particularly promising for two reasons: they are strong Mott insulators with a large *U*/*t* ratio, and their spin–orbit coupling is substantially weaker than that of 4*d* and 5*d* elements. However, only a few suitable systems have been identified to date (see, for example, refs. ^50–54^), and these often exhibit distortions from the ideal pyrochlore lattice. The impact of such distortions on our model would need to be carefully considered.

Our results apply to corner-sharing tetrahedral lattices in general, including the 2D chequerboard lattice^25^ (see Supplementary Information Note F for a study of 16- to 48-site chequerboard systems). Although it is less common than the pyrochlore lattice in real materials, one wonders whether such geometry could emerge in quasi-2D square lattice compounds, possibly mediated by interlayer atoms, or, perhaps, in 2D materials and synthetic quantum platforms. From a theoretical perspective, it would be interesting to investigate in greater detail the robustness of the RVB-liquid behaviour and spin–charge separation to an asymmetry between the nearest-neighbour and next-nearest-neighbour (diagonal) hopping terms on the chequerboard lattice. It would also be interesting to study the effects of non-magnetic vacancy disorder, which has been recently argued could destabilize some but not all short-range RVB liquid phases in 2D, depending on the lattice^55^.

As speculated earlier, the RVB singlet state in our system could exhibit an emergent U(1) gauge symmetry with power-law singlet correlators and gapless (photon) excitations (in three dimensions)^37–39^. In the presence of itinerant (fermionic^56^) holons, this is reminiscent of models used to investigate Fermi-liquid star behaviour^57^ and may provide another experimental avenue to exploring it.

In closing, our work proposes kinetic energy frustration of doped Mott insulators as a route to realizing robust topologically ordered states, in contrast to the conventional approach based on geometrically frustrated interactions. This holds great promise for the theoretical and experimental discovery of emergent states of matter. Any potential connection between kinetically frustrated RVB liquids and superconductivity^4^ remains an open question for future work.

## Methods

We studied a 16-site system consisting of a single conventional cubic unit cell of the pyrochlore lattice (see Supplementary Information Note C for illustrations) using exact diagonalization with the Lanczos method to compute the lowest eigenvalues and corresponding eigenstates in each magnetization sector. We also considered a 32-site pyrochlore system consisting of a 2 × 2 × 2 cell constructed from the fcc primitive lattice vectors. For this system size, we used two-site DMRG with an energy convergence criterion of 10^−10^ and a maximum bond dimension of 12,800. We imposed periodic boundary conditions and created a one-dimensional snake path of the system following the numbering shown in Supplementary Information Note C. The choice of snake path seems to have little impact on the convergence of our highly entangled three-dimensional system. The DMRG calculations were performed using the TeNPy Library (v.0.10.0)^58^.

A single DMRG calculation gives one ground state. When the ground state is degenerate, we biased each ground state and computed correlations as an average over all ground states. For pure hopping (*J* = 0), the full ground-state manifold was found by pinning the unpaired spin by adding an on-site magnetic field. This gave an overcomplete set of ground states. A linearly independent set of ground states was then found from a Gram–Schmidt decomposition. A similar approach was deployed to study our system on the chequerboard lattice, using exact diagonalization for 16 sites (one and two holes) and 24 sites (one hole) and using DMRG for 24 sites (two holes), 36 sites (one and two holes) and 48 sites (one and two holes).

## Online content

Any methods, additional references, Nature Portfolio reporting summaries, source data, extended data, supplementary information, acknowledgements, peer review information; details of author contributions and competing interests; and statements of data and code availability are available at 10.1038/s41567-025-02923-8.

## Supplementary information

Supplementary Information Supplementary Sections A–G and Figs. 1–8.

## Data Availability

All data shown in the figures can be derived using computer simulations, with parameters given in the paper and Supplementary Information.

## Code availability

All code used in this work is readily available through libraries, as discussed in Methods.
