# Supplementary material for: A resonant valence bond spin liquid in the dilute limit of doped frustrated Mott insulators
Source: Nat Phys. 2025 May 29;21(8):1211–6. doi: 10.1038/s41567-025-02923-8 (PMC12343288; doi:10.1038/s41567-025-02923-8)
Supplement: Supplementary file 1 — Supplementary Sections A–G and Figs. 1–8. [file 41567_2025_2923_MOESM1_ESM.pdf]

nature physics

Supplementary information https://doi.org/10.1038/s41567-025-02923-8

A resonant valence bond spin liquid in the dilute limit of doped frustrated Mott insulators

In the format provided by the authors and unedited

Supplementary Material Table of Content

Supp. Mat. Note A: Proofs
Supp. Mat. Note B: The counter-Nagaoka effect
Supp. Mat. Note C: Models and methods
Supp. Mat. Note D: Doping and correlations
Supp. Mat. Note E: Two holes
Supp. Mat. Note F: Checkerboard
Supp. Mat. Note G: Experiments

SM Note A: Proofs

In this section, we present the proof of the first result in our work, relating to the ground state (GS) of Eq. (1) in the main text, for a single hole in the thermodynamic limit when $J = 0$ and $t > 0$ (frustrated hopping). We note that the proof applies generally to lattices of corner-sharing tetrahedra, including the three-dimensional pyrochlore lattice, as well as the two-dimensional checkerboard lattice.

1. Ground state energy

Since the Hamiltonian $\hat{\mathcal{H}}$ includes only nearest neighbour hopping terms, it can be decomposed as a sum of Hamiltonians $\hat{\mathcal{H}}^{\alpha}$ acting on each tetrahedron α:

$$\hat{\mathcal{H}} = \sum_{\alpha} \hat{\mathcal{H}}^{\alpha} \,, \tag{A1}$$

where the dual lattice index α labels the different tetrahedra and $\hat{\mathcal{H}}^{\alpha}$ includes only the six bonds of the tetrahedron α.

The ground state $|\Psi_0\rangle$ can be expressed as

$$|\Psi_0\rangle = \sum_{\boldsymbol{r}} |\phi_{\boldsymbol{r}}\rangle \,, \tag{A2}$$

where $|\phi_{\boldsymbol{r}}\rangle$ represents a state where the holon occupies the site $\boldsymbol{r}$ and the rest of the spins are in an arbitrary spin configuration, $\langle\phi_{\boldsymbol{r}}|\phi_{\boldsymbol{r}'}\rangle = \delta_{\boldsymbol{r},\boldsymbol{r}'}\langle\phi_{\boldsymbol{r}}|\phi_{\boldsymbol{r}}\rangle$, and $\langle\Psi_0|\Psi_0\rangle = 1$.

After introducing the states

$$|\chi_{\alpha}\rangle = \sum_{\boldsymbol{r}\in\alpha} |\phi_{\boldsymbol{r}}\rangle \,, \tag{A3}$$

where the sum runs over the 4 sites of the tetrahedron α, we compute the expectation value of the energy,

$$E_g = \sum_{\boldsymbol{r},\boldsymbol{r}'}\langle\phi_{\boldsymbol{r}}|\hat{\mathcal{H}}|\phi_{\boldsymbol{r}'}\rangle = \sum_{\langle\boldsymbol{r},\boldsymbol{r}'\rangle}\langle\phi_{\boldsymbol{r}}|\hat{\mathcal{H}}|\phi_{\boldsymbol{r}'}\rangle = \sum_{\alpha}\langle\chi_{\alpha}|\hat{\mathcal{H}}^{\alpha}|\chi_{\alpha}\rangle \,, \tag{A4}$$

where $\langle\boldsymbol{r},\boldsymbol{r}'\rangle$ restricts the sum to nearest-neighbour sites. By introducing the probability distribution over tetrahedra:

$$p_{\alpha} = \frac{1}{2}\langle\chi_{\alpha}|\chi_{\alpha}\rangle \,, \tag{A5}$$

where the normalisation condition $\sum_{\alpha} p_{\alpha} = 1$ follows from $\langle\Psi_0|\Psi_0\rangle = 1$, we express E_g as

$$E_g = \sum_{\alpha} p_{\alpha} 2\frac{\langle\chi_{\alpha}|\hat{\mathcal{H}}|\chi_{\alpha}\rangle}{\langle\chi_{\alpha}|\chi_{\alpha}\rangle} \,. \tag{A6}$$

Now, assume that $E_g < -4t$. Since p_{α} is a probability distribution over tetrahedra, this assumption implies that there is at least one tetrahedron β for which

$$2\frac{\langle\chi_{\beta}|\hat{\mathcal{H}}|\chi_{\beta}\rangle}{\langle\chi_{\beta}|\chi_{\beta}\rangle} < -4t \quad \text{or} \quad \frac{\langle\chi_{\beta}|\hat{\mathcal{H}}|\chi_{\beta}\rangle}{\langle\chi_{\beta}|\chi_{\beta}\rangle} < -2t \,. \tag{A7}$$

Eq. (A7) implies that the ground state energy of a single tetrahedron is lower than $-2t$. However, the ground state energy of a single tetrahedron is exactly $-2t$, which implies that E_g cannot be lower than $-4t$.

For completeness, let us close by showing explicitly that the single tetrahedron GS energy is $-2t$. If we consider a system consisting of four fully connected sites, labelled $1, 2, 3, 4$, with one holon and three spins, we can define the wave functions

$$\begin{aligned}
|\Psi^{\rm tet}_{1\sigma}\rangle &= \frac{1}{\sqrt{3}}\hat{c}^{\dagger}_{1\sigma}\left(\hat{d}^{\dagger}_{23} + \hat{d}^{\dagger}_{34} + \hat{d}^{\dagger}_{42}\right)|0\rangle \\
|\Psi^{\rm tet}_{2\sigma}\rangle &= \frac{1}{\sqrt{3}}\hat{c}^{\dagger}_{2\sigma}\left(\hat{d}^{\dagger}_{13} + \hat{d}^{\dagger}_{34} + \hat{d}^{\dagger}_{41}\right)|0\rangle \\
|\Psi^{\rm tet}_{3\sigma}\rangle &= \frac{1}{\sqrt{3}}\hat{c}^{\dagger}_{3\sigma}\left(\hat{d}^{\dagger}_{12} + \hat{d}^{\dagger}_{24} + \hat{d}^{\dagger}_{41}\right)|0\rangle \,,
\end{aligned} \tag{A8}$$

with $\hat{d}^{\dagger}_{ij} = \frac{1}{\sqrt{2}}(\hat{c}^{\dagger}_{i\uparrow}\hat{c}^{\dagger}_{j\downarrow} - \hat{c}^{\dagger}_{i\downarrow}\hat{c}^{\dagger}_{j\uparrow})$. Note that $\hat{d}^{\dagger}_{ij} = \hat{d}^{\dagger}_{ji}$. The state $|\Psi^{\rm tet}_{i\sigma}\rangle$ has a static spin σ on site i, while the holon and a singlet resonate on the remaining three sites. Consequently, the total spin of the state is σ. Applying the single tetrahedron Hamiltonian to this state, we find that hopping to site i indeed is cancelled, and the states are eigenstates with energy $-2t$. Note that the state where site 4 has a static spin is a superposition of the three states in Eq. (A8): $|\Psi^{\rm tet}_{4\sigma}\rangle = \frac{1}{\sqrt{3}}\hat{c}^{\dagger}_{4\sigma}\left(\hat{d}^{\dagger}_{12} + \hat{d}^{\dagger}_{23} + \hat{d}^{\dagger}_{31}\right)|0\rangle = -(|\Psi^{\rm tet}_{1\sigma}\rangle + |\Psi^{\rm tet}_{2\sigma}\rangle + |\Psi^{\rm tet}_{3\sigma}\rangle)$.

2. Ground state wave function

Consider now the resonant valence bond (RVB) state:

$$|\Psi_{\rm RVB}\rangle = \frac{1}{\sqrt{\kappa N}}\sum_{\boldsymbol{r}} |\phi^{\rm RVB}_{\boldsymbol{r}}\rangle \,, \tag{A9}$$

with

$$|\phi_{\boldsymbol{r}}^{\mathrm{RVB}}\rangle = \sum_{a_{\boldsymbol{r}}} |\Psi_{a_{\boldsymbol{r}}}\rangle, \quad \kappa = \langle\phi_{\boldsymbol{r}}^{\mathrm{RVB}}|\phi_{\boldsymbol{r}}^{\mathrm{RVB}}\rangle\,, \tag{A10}$$

where $N \to \infty$ is the number of sites, the index $a_{\boldsymbol{r}}$ runs over all dimer coverings with one dimer per tetrahedron on the lattice with the site $\boldsymbol{r}$ excluded and $|\Psi_{a_{\boldsymbol{r}}}\rangle$ is the corresponding direct product of singlet states on each dimer:

$$|\Psi_{a_{\boldsymbol{r}}}\rangle = \prod_{\langle i,j\rangle \in a_{\boldsymbol{r}}} \hat{d}_{ij}^{\dagger}|0\rangle\,. \tag{A11}$$

Substituting Eq. (A11) into Eq. (A10), and thence into Eq. (A9), we obtain

$$|\Psi_{\mathrm{RVB}}\rangle = \frac{1}{\sqrt{\kappa N}} \sum_{\boldsymbol{r},\{a_{\boldsymbol{r}}\}} |\Psi_{a_{\boldsymbol{r}}}\rangle = \frac{1}{\sqrt{\kappa N}} \sum_{\boldsymbol{r},\{a_{\boldsymbol{r}}\}} \prod_{\langle i,j\rangle \in a_{\boldsymbol{r}}} \hat{d}_{ij}^{\dagger}|0\rangle\,. \tag{A12}$$

From Eq. (A4), we see that

$$E_{\mathrm{RVB}} \equiv \langle\Psi_{\mathrm{RVB}}|\hat{\mathcal{H}}|\Psi_{\mathrm{RVB}}\rangle = \frac{1}{\kappa N}\sum_{\alpha}\langle\chi_{\alpha}^{\mathrm{RVB}}|\hat{\mathcal{H}}^{\alpha}|\chi_{\alpha}^{\mathrm{RVB}}\rangle, \tag{A13}$$

with

$$|\chi_{\alpha}^{\mathrm{RVB}}\rangle = \sum_{\boldsymbol{r}\in\alpha} |\phi_{\boldsymbol{r}}^{\mathrm{RVB}}\rangle\,. \tag{A14}$$

According to Eq. (A10),

$$|\phi_{\boldsymbol{r}}^{\mathrm{RVB}}\rangle = |\tilde{\Psi}_{\boldsymbol{r}}^{\boldsymbol{r}_1\boldsymbol{r}_2}\rangle + |\tilde{\Psi}_{\boldsymbol{r}}^{\boldsymbol{r}_2\boldsymbol{r}_3}\rangle + |\tilde{\Psi}_{\boldsymbol{r}}^{\boldsymbol{r}_1\boldsymbol{r}_3}\rangle\,, \tag{A15}$$

where $\boldsymbol{r}_1, \boldsymbol{r}_2, \boldsymbol{r}_3$ denote the 3 sites of the tetrahedron α that are not occupied by the holon (the pair $\boldsymbol{r}_i\boldsymbol{r}_j$ is equivalent to $\boldsymbol{r}_j\boldsymbol{r}_i$) and

$$|\tilde{\Psi}_{\boldsymbol{r}}^{\boldsymbol{r}_i\boldsymbol{r}_j}\rangle = \sum_{a_{\boldsymbol{r}}/d\in(\boldsymbol{r}_i,\boldsymbol{r}_j)} |\Psi_{a_{\boldsymbol{r}}}\rangle\,, \tag{A16}$$

where the sum runs over dimer coverings $|\Psi_{a_{\boldsymbol{r}}}\rangle$ with one holon at site $\boldsymbol{r}$ and one singlet on the bond $(\boldsymbol{r}_i\boldsymbol{r}_j)$ with $1 \leq i,j \leq 3$ and $i < j$. By inserting Eq. (A15) in Eq. (A14), we obtain:

$$|\chi_{\alpha}^{\mathrm{RVB}}\rangle = \sum_{\boldsymbol{r}\in\alpha, i<j} |\tilde{\Psi}_{\boldsymbol{r}}^{\boldsymbol{r}_i\boldsymbol{r}_j}\rangle\,. \tag{A17}$$

The sum contains 12 terms corresponding to the 12 different ways of allocating one holon (in one of the 4 sites of the tetrahedron) and one singlet in one of the 3 pairs that can be formed with the 3 remaining sites. We will group these 12 terms in 4 groups, where each group consists of the sum of three states:

$$|\tilde{\Psi}_{\boldsymbol{r}}^{\alpha}\rangle = |\tilde{\Psi}_{\boldsymbol{r}_k}^{\boldsymbol{r}_i\boldsymbol{r}_j}\rangle + |\tilde{\Psi}_{\boldsymbol{r}_j}^{\boldsymbol{r}_k\boldsymbol{r}_i}\rangle + |\tilde{\Psi}_{\boldsymbol{r}_i}^{\boldsymbol{r}_j\boldsymbol{r}_k}\rangle\,. \tag{A18}$$

The coordinates $\boldsymbol{r}_i, \boldsymbol{r}_j, \boldsymbol{r}_k$ denote the 3 distinct sites of the tetrahedron (one containing the holon and the other two forming a singlet state) that are *different* from the 'passive' site $\boldsymbol{r}$. We note that when considering only the four sites on the tetrahedron α, $|\tilde{\Psi}_{\boldsymbol{r}}^{\alpha}\rangle$ correspond exactly to the eigenstates of the single tetrahedron where one site is passive and the three remaining sites contain a holon and singlet resonating.

Next, if we denote the coordinates of the 4 sites of the tetrahedron α by $\boldsymbol{r}_a$ with $1 \leq a \leq 4$, we have:

$$|\chi_{\alpha}^{\mathrm{RVB}}\rangle = |\tilde{\Psi}_{\boldsymbol{r}_1}^{\alpha}\rangle + |\tilde{\Psi}_{\boldsymbol{r}_2}^{\alpha}\rangle + |\tilde{\Psi}_{\boldsymbol{r}_3}^{\alpha}\rangle + |\tilde{\Psi}_{\boldsymbol{r}_4}^{\alpha}\rangle\,, \tag{A19}$$

and

$$\begin{aligned} E_{\mathrm{RVB}} &= \frac{1}{\kappa N}\sum_{\alpha}\langle\chi_{\alpha}^{\mathrm{RVB}}|\hat{\mathcal{H}}^{\alpha}|\chi_{\alpha}^{\mathrm{RVB}}\rangle \\ &= \frac{1}{\kappa N}\sum_{\alpha}\sum_{a,b=1}^{4}\langle\tilde{\Psi}_{\boldsymbol{r}_a}^{\alpha}|\hat{\mathcal{H}}^{\alpha}|\tilde{\Psi}_{\boldsymbol{r}_b}^{\alpha}\rangle\,. \end{aligned} \tag{A20}$$

Finally, we note that

$$\hat{\mathcal{H}}^{\alpha}|\tilde{\Psi}_{\boldsymbol{r}_a}^{\alpha}\rangle = -2t\,|\tilde{\Psi}_{\boldsymbol{r}_a}^{\alpha}\rangle\,, \tag{A21}$$

implying that

$$\begin{aligned} E_{\mathrm{RVB}} &= -\frac{2t}{\kappa N}\sum_{\alpha}\sum_{a,b=1}^{4}\langle\tilde{\Psi}_{\boldsymbol{r}_a}^{\alpha}|\tilde{\Psi}_{\boldsymbol{r}_b}^{\alpha}\rangle \\ &= -\frac{2t}{\kappa N}\sum_{\alpha}\langle\chi_{\alpha}^{\mathrm{RVB}}|\chi_{\alpha}^{\mathrm{RVB}}\rangle \\ &= -\frac{4t}{\kappa N}\sum_{\boldsymbol{r}}\langle\phi_{\boldsymbol{r}}^{\mathrm{RVB}}|\phi_{\boldsymbol{r}}^{\mathrm{RVB}}\rangle = -4t\,. \end{aligned} \tag{A22}$$

Since we have shown that $-4t$ is a strict lower bound for the energy, Eq. (A22) implies that $|\Psi_{\mathrm{RVB}}\rangle$ is an exact ground state of $\hat{\mathcal{H}}$. Eq. (A21) clarifies the name 'passive' for the site $\boldsymbol{r}$: since $|\tilde{\Psi}_{\boldsymbol{r}}^{\alpha}\rangle$ is an eigenstate of $\hat{\mathcal{H}}^{\alpha}$, the holon never visits that site because of the destructive interference produced by the linear superposition (A18).

3. Considerations about the thermodynamic limit

The proof holds in the thermodynamic limit, as only in that limit can we accommodate one dimer in each tetrahedron. Inserting a hole in a finite lattice with an integer number of tetrahedra leads to an unpaired spin that can be associated with a spinon. In this scenario, the dimer coverings are not complete because there is one tetrahedron which does not contain a dimer. We can always choose that tetrahedron to be located at the boundary of the finite lattice and also to be the one containing the unpaired spin. Since the boundary disappears upon taking the thermodynamic limit, the unpaired spin and the missing dimer become irrelevant in this limit. However, this simple observation has two important implications. Firstly, as expected for an RVB ground state, the spin and the charge of the fermion added to the Mott insulator separate, i.e., the probability of finding the holon

at any finite distance from the spinon is zero in the thermodynamic limit. Secondly, on a finite size lattice the spinon must necessarily occupy a passive site of the tetrahedron, implying that the holon will never visit the site occupied by the unpaired spin. In other words, the spinon is expected to have infinite mass (static spinon) and the spinon-holon interaction is expected to be repulsive. Both observations are supported by our numerical results.

4. Proposed finite-size ground state wave function

From numerics (discussed in Supp. Mat. Notes C and D), we find that the one-hole GS energy $-4t$ can be met in finite size systems and even when the unpaired spin is fixed at a given site; in the latter case the GS is unique. This allows us to conjecture a form for the wave function that is given by the equal amplitude superposition of all dimer-singlet states that have a spin fixed and a delocalised holon, subject to the constraint of no more than one dimer per tetrahedron. Remarkably, we find that the conjectured state is identical within numerical accuracy to the ground state found for the 16-site system using ED, and to the 32-site system using DMRG. We also find this to be the case for the two-holes ground state (i.e., the equal amplitude superposition of all dimer-singlet states that have two delocalised holons, subject to the constraint of no more than one dimer per tetrahedron). We therefore propose that such GS wave functions are in fact exact for all system sizes.

Note that any dimer configuration with a fixed spin and a hole (or two holes) necessarily has one less dimer than tetrahedra. Under the condition of no more than one dimer per tetrahedron, this means that there is precisely one tetrahedron without dimers in the configuration. Notably, we find that the proposed GS wave function is invariant to fixing the location of this tetrahedron.

5. Vanishing wave function in the stoichiometric limit

Curiously, we find that our problem does not admit a straightforward stoichiometric limit of vanishing hole density. If we take the equal amplitude superposition of all dimer-singlet states on the pyrochlore lattice, without holons or unpaired spins, the resulting wave function vanishes identically for both the 16-site and the 32-site system. This surprising result – which to the best of our knowledge had not been pointed out in the literature before – holds irrespective of whether we impose the constraint of one and only one dimer per tetrahedron (relevant to our RVB liquid state) or we consider all possible dimer configurations in general [1].

SM Note B: The counter-Nagaoka effect

Itinerant holons in large-U Hubbard models near half-filling are known to influence the spin correlations. For example, Nagaoka's theorem [2, 3] shows that a single hole in the absence of interactions between the spins is capable of inducing a ferromagnetic ground state. The phenomenon is rooted in the fact that spin correlations affect the kinetic energy of the holon. In Nagaoka's case, a ferromagnetic pattern allows for perfect constructive interference of the holon world lines, thus minimising the kinetic energy. The effect is remarkably stable (namely, a finite magnetisation persists) up to 20% hole doping [4–7] (however, see also Ref. 8 for considerations about a possible breakdown mechanism due to phase separation).

More recently, Haerter and Shastry [9] pointed out that the same kinetic mechanism can give rise to strikingly different spin correlations – namely, weak metallic antiferromagnetism – if the electronic lattice is frustrated; this occurs, for instance, when the electron hopping amplitude is negative, and the elementary loops on the lattice encompass an odd number of bonds (see also Ref. 10 for earlier results hinting at this phenomenon). This counterpart to the Nagaoka effect was further studied in Refs. 11 and 12 and later dubbed the 'counter-Nagaoka effect' [13].

The nature of the state on the frustrated triangular lattice was investigated numerically [11, 12, 14] and shown to exhibit correlations that depart significantly from the behaviour expected for the quantum Heisenberg antiferromagnet, towards a more classical nature (although not entirely: a small deviation from classical correlations is observed [11]). This work also demonstrated that the kinetic energy mechanism was akin to a finite spin interaction term proportional to the hole density (namely, $J_t \sim t/N$, where N is the number of lattice sites, in the presence of a single hole).

The effect has been recently investigated in ladders in Ref. 14; and on a Husimi cactus of triangles (akin locally to a kagome lattice) where it was shown to form a regular valence bond pattern [15].

To date, studies of the counter-Nagaoka effect have focused on 2D (or quasi-1D) systems, where it is found to lead to magnetically ordered low-energy states. In this work, we explore the behaviour on the 3D pyrochlore lattice (see Fig. 1 in the main text), renowned for its strong frustration effects (and our results straightforwardly apply also to a planar arrangement of tetrahedra, also known as the 2D checkerboard lattice).

SM Note C: Models and methods

In the main text, we expressed the Hamiltonian for our system in terms of constrained electron operators for notational convenience. The constraint can be made explicit by substituting $\hat{c}_{j\sigma} \to \hat{c}_{j\sigma}\hat{P}_{j\sigma}$, where on the right hand side we have the conventional femionic annihilation operator, and $\hat{P}_{i\sigma} = (1 - \hat{n}_{i\bar{\sigma}})$ is the projection operator

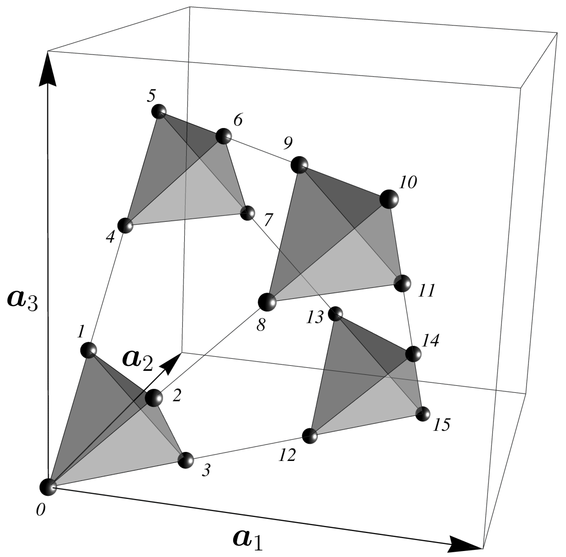

FIG. S1. *16-site pyrochlore system.* This is constructed from a single cubic unit cell with lattice vectors $\boldsymbol{a}_1 = (1,0,0)$, $\boldsymbol{a}_2 = (0,1,0)$ and $\boldsymbol{a}_3 = (0,0,1)$.

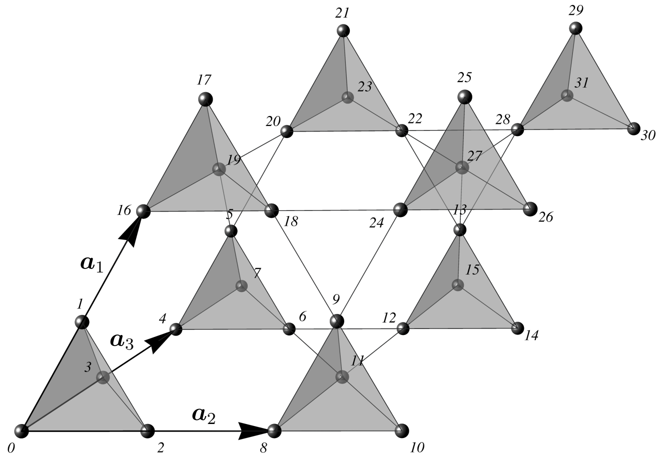

FIG. S2. *32-site pyrochlore system.* This is constructed from a $2 \times 2 \times 2$ lattice of the ffc unit cell with lattice vectors $\boldsymbol{a}_1 = (0,1/2,1/2)$, $\boldsymbol{a}_2 = (1/2,0,1/2)$ and $\boldsymbol{a}_3 = (1/2,1/2,0)$. Each unit cell has four sites (a single tetrahedron).

that ensures no double occupancy, with $\hat{n}_{i\sigma}$ being the customary density operator. This leads to the Hamiltonian written as

$$\hat{\mathcal{H}} = -t \sum_{\langle i,j \rangle} \sum_{\sigma} \left[\hat{P}_{i\sigma} \hat{c}^{\dagger}_{i\sigma} \hat{c}_{j\sigma} \hat{P}_{j\sigma} + h.c. \right] + J \sum_{\langle i,j \rangle} \hat{\boldsymbol{S}}_i \cdot \hat{\boldsymbol{S}}_j \,, \quad \text{(C1)}$$

where once again $\langle i,j \rangle$ denotes nearest-neighbour pairs of sites; $\hat{c}^{\dagger}_i$ ($\hat{c}_i$) is the fermionic creation (annihilation) operator at site i; and $\hat{\boldsymbol{S}}_i$ is the corresponding spin operator, $\hat{\boldsymbol{S}}_i = \frac{1}{2} \sum_{\sigma,\sigma'} \hat{c}^{\dagger}_{i\sigma} \boldsymbol{\sigma}_{\sigma\sigma'} \hat{c}_{i\sigma'}$.

We study 16- and 32-site systems of the pyrochlore lattice, both with periodic boundary conditions. The 16-site system is a conventional cubic unit cell, illustrated

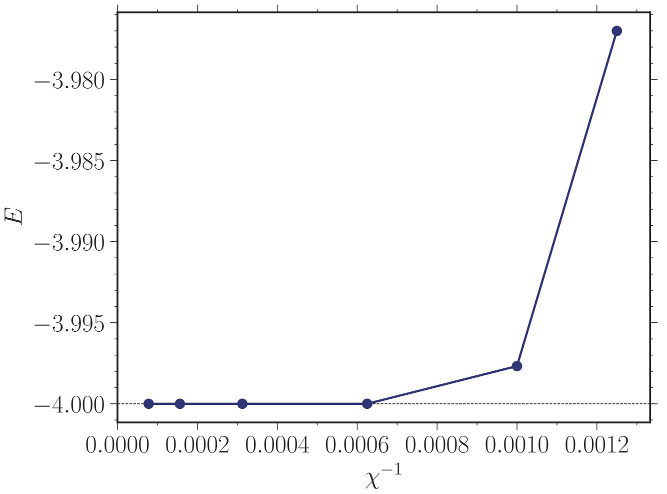

FIG. S3. *Energy as a function of the inverse bond dimension for the 32-site pyrochlore system.* The bond dimensions used are $\chi = \{800, 1000, 1600, 3200, 6400, 12800\}$.

in Fig. S1. The 32-site system is a $2 \times 2 \times 2$ spherical cell constructed from the fcc primitive lattice vectors, illustrated in Fig. S2.

The 16-site and 32-site systems are chosen as they respect the symmetries of the pyrochlore lattice. Other shapes of the unit cell show a tendency to seemingly spurious finite-size effects at very low temperatures in a way akin to partial order-by-disorder effects induced by shape anisotropy. The next possible symmetric system sizes would be the $3 \times 3 \times 3$ spherical fcc system, with 108 sites, or a $2 \times 2 \times 2$ cubic system with 128 sites. However, these system sizes are beyond the computational reach of our study.

For the 16-site system, we perform ED with Lanczos to compute the 800 smallest eigenvalues and corresponding eigenstates in each magnetisation sector. We ensure we capture all ground states by checking that their number stays the same when the number of computed eigenvalues and eigenstates is doubled.

We use two-site DMRG to study the 32-site system, with an energy convergence criterion of 10^{-10} and a maximum bond dimension up to 12800. This is sufficient to reach convergence, as shown in Fig. S3. Using periodic boundary conditions, we create a 1D snake path of the system that follows the numbering in Fig. S2. The choice of snake path seems to have little impact on the convergence for our highly entangled 3D system. The DMRG calculations were performed using the TeNPy Library (version 0.10.0) [16].

A single DMRG calculation gives one ground state. When the ground state is degenerate due to an odd number of spins, we bias each ground state and compute correlations as an average over all ground states. In the case of pure hopping ($J = 0$, $t = 1$), the full GS manifold is found by pinning the unpaired spin by adding an on-site magnetic field. This gives an overcomplete set of ground

states, and a linearly independent set of ground states is then found via Gram-Schmidt decomposition.

For the studies of the classical Heisenberg model in Supp. Mat. Note D, we use Monte Carlo (MC) simulations with the Metropolis algorithm [17]. The classical structure factors are computed after equilibrating the system down to $\beta = 10^3$. In order to compare quantitatively classical spins with quantum spin $S = 1/2$ degrees of freedom, we tune the classical spin length to satisfy $S^2 = 3/4$.

As mentioned in the main text, our results hold for lattices of corner sharing tetrahedra and therefore also for the 2D checkerboard lattice. Indeed, the 16-site unit cell of the 2D checkerboard lattice ($4 \times 2 \times 2$) is identical to the one for the pyrochlore lattice ($16 \times 1 \times 1 \times 1$). In our work, we further consider a 24-site ($4 \times 3 \times 2$) checkerboard lattice using ED (1 hole) and DMRG (2 holes) as well as 36- ($4 \times 3 \times 3$) and 48-site ($4 \times 4 \times 3$) checkerboard lattices with 1 and 2 holes using DMRG.

SM Note D: Doping and correlations

In the first instance, we consider the case of non-interacting spins ($t = 1$, $J = 0$) at zero temperature in the presence of a single hole. For all system sizes, we find that the ground state energy bound is met at $-4t$. For 16 and 32 sites, the GS degeneracy is 18 and 34, respectively.

An overall 2-fold degeneracy can be readily understood from the odd number of spin-1/2 degrees of freedom in the system. The residual degeneracy is clearly related to the presence of an unpaired spin. Indeed, we find a unique ground state when the unpaired spin is pinned to a given lattice site, resulting in a set of 16 and 32 linearly-dependent states that span the GS manifold. A set of orthonormal GS wave functions can then be found using Gram-Schmidt decomposition, for example, giving the 9-fold and 17-fold degeneracy. This is in contrast with the behaviour observed for two holes (see Supp. Mat. Note E), where there are no unpaired spins and the GS is unique. While the specific degeneracy of the single-hole ground state for arbitrary system sizes remains to be understood, our results suggest that the scaling is at most linear in system size.

The holon density shows weak repulsive dependence on the distance from the fixed spin, as illustrated in Fig. 2 in the main text. Correspondingly, the expectation value of the hole hopping operator has a suitably inverse dependence, leading to the exact Hamiltonian eigenvalue $-4t$. This is confirmed by computing the expectation value $\langle \sum_{\sigma,j} \hat{c}^{\dagger}_{i,\sigma} \hat{c}_{j,\sigma} \rangle$, where j runs over all nearest-neighbour sites of i. Within numerical accuracy, we find indeed that this expectation value is precisely $4\langle 1 - \hat{n}_i \rangle$ for all sites i, where $\langle 1 - \hat{n}_i \rangle$ is the holon density at site i.

We further checked that, within numerical precision, the expectation value of the projector onto the state with maximum angular momentum for each tetrahedron with no holon,

$$Q_{\rm tet} = \left\langle \hat{\boldsymbol{S}}^2_{\rm tet} \left(\hat{\boldsymbol{S}}^2_{\rm tet} - 2 \right) \left(\sum_{i \in {\rm tet}} \hat{n}_i - 3 \right) \right\rangle , \qquad \text{(D1)}$$

vanishes identically in the ground state for all tetrahedra. Here, $\hat{\boldsymbol{S}}_{\rm tet} = \sum_{i \in {\rm tet}} \hat{\boldsymbol{S}}_i$ is the total spin of the given tetrahedron; and $\sum_{i \in {\rm tet}} \hat{n}_i$ takes on the value 4 or 3 depending on whether a holon is absent or present on the tetrahedron, respectively.

We find within numerical accuracy that the GS wave function (with a fixed spin) from 16-site ED and 32-site DMRG for the pyrochlore lattice, and 16- and 24-site ED for the checkerboard lattice , is identical to a state constructed as the equal amplitude superposition of dimer-singlet states with a holon and the further constraint of no more than one dimer per tetrahedron.

We compute the spin structure factor of our RVB liquid state, averaged over all ground states of our system for $t = 1$ and $J = 0$, as $\mathcal{S}(\boldsymbol{q}) = \sum_{i,j} \langle S^z_i S^z_j \rangle e^{-i\boldsymbol{q}\cdot(\boldsymbol{r}_i - \boldsymbol{r}_j)}/N$, where $\boldsymbol{r}_i$ is the position of site i. This is shown in Fig. S4 (upper panels) for the 32-site system, obtained using DMRG. There is indeed no evidence of symmetry breaking. While one can recognise features akin to those of the spin-1/2 quantum Heisenberg model (obtained using DMRG; see Fig. S4, middle panels), the doping-induced correlations in the structure factor of the RVB liquid state in our work are clearly distinct in a way that promises to be measurable in experiments. We also compare it for reference to the case of a classical Heisenberg AFM obtained using classical MC simulations (Fig. S4, lower panels). Both the quantum and classical pyrochlore Heisenberg AFM are known to be fully frustrated and in a spin liquid GS [18]. In the case of the pure Heisenberg models we do not introduce any holes and we scale the resulting correlations by $(N - N_h)/N$, where $N = 32$ and $N_h = 1$ are the number of sites and holes in the RVB state, respectively.

SM Note E: Two holes

We compute the GS in the presence of two holes and study the GS spin and holon correlations for $t = 1$ and $J = 0$. We find that the GS is unique with energy $-8t$ in the $S^z = 0$ sector for both the 16-site and 32-site systems, which is exactly twice the GS energy of a single hole.

We verified that, within numerical accuracy, the GS wave function obtained with ED for 16 sites and DMRG for 32 sites is identical to the equal amplitude superposition of all dimer-singlet states hosting two holons, subject to the constraint of no more than one dimer per tetrahedron. (We also confirmed that this result holds for the 24-site checkerboard lattice.)

We find that the doping-induced spin correlations when two holes are present (see Fig. 2 in the main text) are similar to the ones obtained for a single hole. Quan-

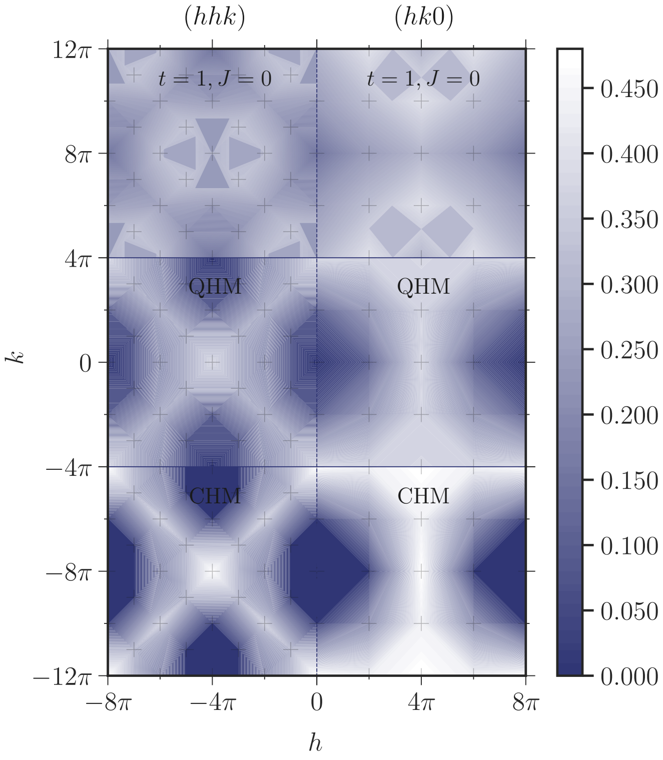

FIG. S4. *Spin structure factors of the 32-site systems.* Top: Doping-induced, 1 hole ($t = 1$ and $J = 0$). Middle: Quantum Heisenberg model (QHM). Bottom: Classical Heisenberg model (CHM). Both Heisenberg models have no holes and their correlations are correspondingly scaled by $(N-1)/N$. Accessible momenta are marked by grey pluses.

titatively, we compare them using the measure:

$$D = \frac{1}{N}\sum_{\boldsymbol{q}}\left[\mathcal{S}_{1-\text{hole}}(\boldsymbol{q}) - \mathcal{S}_{2-\text{holes}}(\boldsymbol{q})\right]^2 , \qquad \text{(E1)}$$

and we find values $\lesssim 10^{-4}$ and $\lesssim 10^{-5}$ for the 16-site and 32-site case, respectively, when $\mathcal{S}_{1-\text{hole}}$ and $\mathcal{S}_{2-\text{holes}}$ are suitably rescaled by $(N - N_h)/N$ to account for the different total number of spins.

Next, we study the effect of having a finite J when two holes are present in the 16-site system. In Fig. S5 we show the GS spin structure factor as a function of J/t with $t = 1$. We find that the spin liquid behaviour persists down to a finite negative value of $(J/t)_c \simeq -0.173$ (hole density 12.5%). This shows that also in the presence of two holes, quantum spin liquid behaviour can be induced over magnetic ordering by frustrated hole doping.

Finally, we study the holon-holon correlations. The dependence on distance between the two holes is shown in Fig. 2 in the main text, for $t = 1$ and $J = 0$ and system sizes 16 and 32 (and, consistently with the structure of the wave function, it overlaps with the spinon-holon correlation for a single-hole GS).

For the 16-site system, we also study the behaviour in presence of interactions, as a function of J/t, in Fig. S6. Note that because of the small system size, we only have

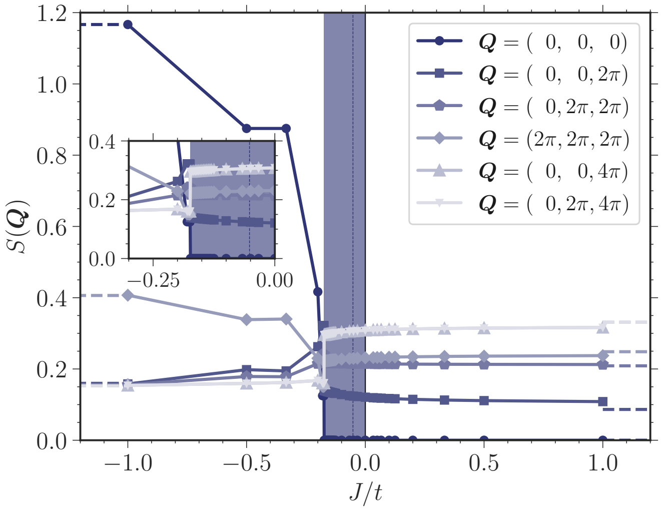

FIG. S5. *Dependence of the ground state spin structure factor on J/t ($t > 0$).* This is shown for all individual reciprocal lattice points for the 16-site system with two holes (density 1/8). Dashed horizontal lines show the $t = 0$ limits, for reference. The shaded region denotes correlations that are antiferromagnetic in spite of a ferromagnetic interaction J, extending down to $(J/t)_c \simeq -0.173$. For comparison, the dashed vertical line inside the shaded region shows the corresponding extent for hole density 1/16, $(J/t)_c \simeq -0.052$ (see Fig. 3 in the main text).

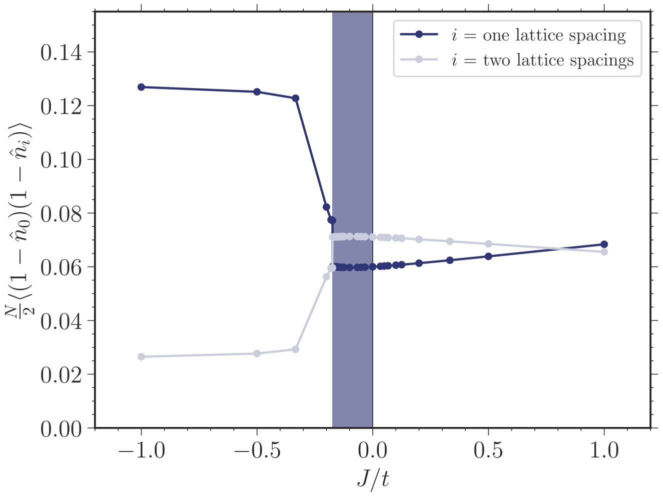

FIG. S6. *Holon-holon correlations as a function of J/t.* $\hat{n}_i$ is the spin density operator on site i. We measure the correlations between a holon at site 0 and a holon at a site either one or two lattice spacings away. The shaded region shows the range of J/t where the spin correlations are antiferromagnetic in spite of a ferromagnetic interaction J.

access to three distances (with periodic boundary conditions, two sites in this system are either one or two lattice spacings apart). Additionally the second-nearest neighbour and third-nearest neighbour correlators give the same values, and we therefore only have two independent values of the correlator. Within the limitation

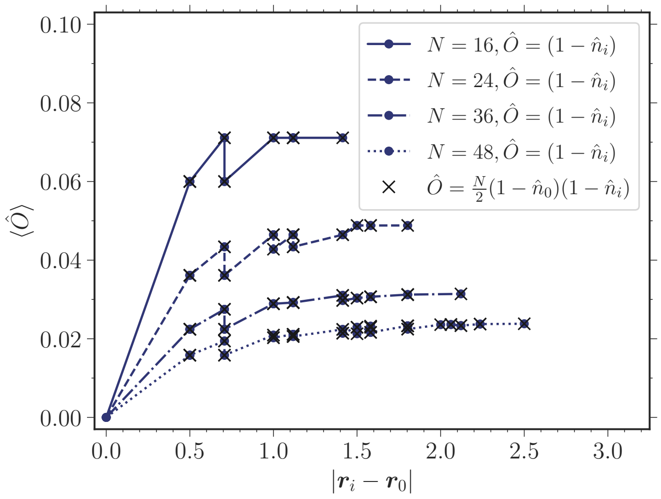

FIG. S7. *Holon correlations on the checkerboard lattice.* The dark-blue dots show the holon density for a checkerboard lattice of 16, 24, 36 and 48 sites, with $t = 1$ and $J = 0$ doped with a single hole, as a function of distance from a pinned spin, indicating spinon-holon deconfinement. Crosses show the holon-holon correlations in equivalent systems with two holes; they are numerically identical to the holon-spinon correlations.

of the small system size accessed in our study, we observe no sign of holon attraction (rather a small tendency to holon repulsion) in the RVB liquid phase. In the large-J ferromagnetic regime, when the GS becomes partially magnetised, holon attraction is introduced by the holons sharing minority spins [13].

SM Note F: Checkerboard

In Fig. S7, we show the spinon-holon correlations for checkerboard systems of 16, 24, 36, and 48 sites, with one hole and a pinned spin ($t = 1$ and $J = 0$). We include in the figure the holon-holon correlations when two holes are present, for the same system sizes. Once again, we find that the holon-holon correlations exactly match the spinon-holon correlations, consistently with our ground state wave function ansatz. Indeed, as already mentioned earlier, we find that the numerically obtained GS wave function for the 16- and 24-site checkerboard lattice is identical within numerical accuracy to a state constructed as the equal amplitude superposition of dimer-singlet states with one or two delocalised holons (with a fixed spin in the one-holon case) and the further constraint of no more than one dimer per tetrahedron.

For a checkerboard system of 36-sites and two holes ($t = 1$ and $J = 0$), we also show in Fig S8 the spin structure factor, for reference.

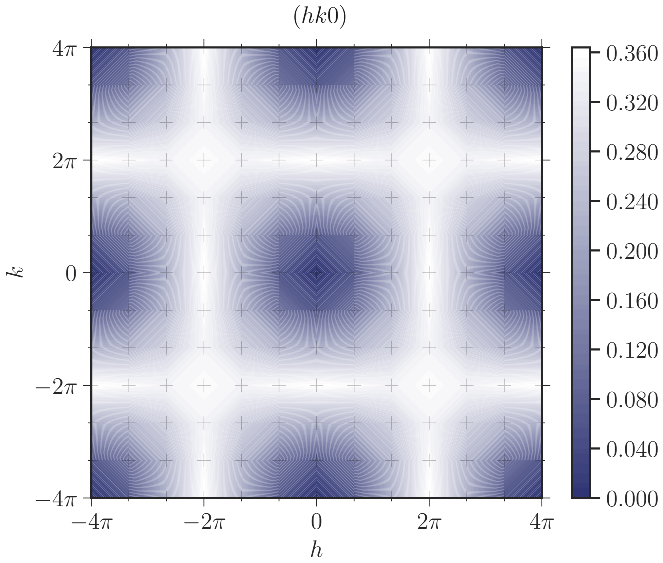

FIG. S8. *Spin structure factor for the 36-site checkerboard lattice.* This is shown for $t = 1$ and $J = 0$, doped with two holes. Accessible momenta are marked by grey pluses.

SM Note G: Experiments

Among the known pyrochlore materials, the platinum group (Pt, Pd, Rh, Ru, Ir, and Os) of compounds, particularly the iridates and ruthenates, have special interest due to their mobile charge carriers and strong spin-orbit coupling [19, 20]. As discussed in the main text, iridate pyrochlore oxides ($RE_2Ir_2O_7$, RE = rare earth) are especially promising because – while being far from a spin-1/2 half-filling Hubbard model description – stoichiometric Ir^{4+} ions have $5d^5$ shells with effective $J_{\text{eff}} = 1/2$ ground state doublets, whereas doping-induced Ir^{5+} have $5d^4$ shells and $J_{\text{eff}} = 0$. In these materials, the iridium ions sit on a lattice of corner-sharing tetrahedra and undergo an antiferromagnetic transition to a long-range ordered state that is characterised by an all-in-all-out (AIAO) arrangement in which the iridium moments point directly into/out of neighbouring tetrahedra [21–23]. The ordering temperature of the Ir sublattice depends on Ir–O–Ir bond angles and hence is highly sensitive to the size of the RE ion [24]. As such, to test our predictions effectively, holes should be introduced while perturbing the IrO_6 octahedra as little as possible. In addition, any obfuscating effects of magnetism from the RE sublattice should be avoided. This could be achieved via the synthesis of various doping series in which non-magnetic RE ions (e.g., Y, La, Lu) are successively substituted by an ion of similar size but different charge (e.g., Ca or Sr).

1. Measurements

Experimentally, the primary indicator of the onset of spin liquid behaviour in the pyrochlore iridates would be

the suppression of the AIAO magnetic order on the Ir sublattice as a function of hole doping. In the materials with non-magnetic RE ions, this can be readily observed in the temperature dependence of the magnetic susceptibility. The onset of AIAO order is typically accompanied by a metal-to-insulator transition [25], and resistivity measurements may also be used to track its suppression (with the caveat that a separation of the magnetic and resistive transitions is also possible, as recently observed in $Sm_2Ir_2O_7$ under applied pressure [26]). Other measurements could be undertaken that provide information on the nature of any observed magnetic ordering, including resonant X-ray scattering, muon spin-relaxation and neutron diffraction [23, 24, 27]. Crystal structure determination and careful modelling would also need to be performed to disentangle any effects on the magnetic properties arising from structural changes or from the increase in disorder caused by the introduction of random defects, from those anticipated due to the addition of holes.

As discussed earlier, some studies of this kind have already been performed, including measurements of polycrystalline samples of $Y_{2-x}Ca_xIr_2O_7$ for $x = 0$–0.2 [28, 29]. Using X-ray photoelectron spectroscopy, Ref. 29 finds that the amount of Ir^{5+} in the sample does indeed increase with Ca doping and, at the same time, transport measurements indicate that the conductivity improves. Moreover, a feature in the resistivity that might be associated with the metal-insulator transition seen in other pyrochlore iridates moves to lower temperatures. And temperature-dependent magnetic susceptibility measurements show that the bifurcation of the zero-field and field-cooled traces (which likely arises due to weak ferromagnetic behaviour and is typically associated with the onset of AIAO order in the pyrochlore iridates [30]), shifts up in temperature with doping. This last result, however, is in contradiction to Ref. 28, which finds that the bifurcation moves to lower temperatures over the same doping range. That study also finds an enhancement of the conductivity as x increases, but no features related to any metal-insulator transition could be discerned. One drawback of these studies is the polycrystalline nature of the samples. Broken symmetry at the surface and the unknown effects of intergrain boundaries in pressed pellets are known to affect the experimental results in these materials, particularly transport measurements, and tend to obscure what is happening in the bulk [26]. So, while a marked effect on the magnetic and transport properties of pyrochlore iridates has already been observed on doping with holes, measurements of high-quality single crystals are necessary to understand these changes and test the predictions discussed here. This requires further investigation into improving synthesis techniques.

2. Crystal growth

Polycrystalline samples of pyrochlore iridates ($RE_2Ir_2O_7$) and ruthenates ($RE_2Ru_2O_7$) are typically synthesised by conventional solid state sintering between 950°C and 1100°C in air using high purity ($> 99.99\%$) oxide or nitrate chemicals [31, 32]. However, in the case of platinum pyrochlores ($RE_2Pt_2O_7$), the low decomposition temperature of PtO_2 around 450°C makes the conventional ceramic-route synthesis impossible. High-pressure (30–40 kbar) and high-temperature (700–1200°C) routes have been used to synthesise polycrystalline and small single crystals of platinum pyrochlore materials [33]. The majority of the platinum group compounds belong to the pyrochlore structure with space group Fd$^-$3m (no.277). Since these compounds decompose at high temperature due to high vapour pressure, single crystals cannot be grown using any melt techniques, such as floating-zone or Bridgman. Instead, successful growth of small crystals have been reported via flux methods using KF and CsCl fluxes at 1100°C [34, 35] (see also our own sample in Fig. 1 in the main text). Recently, high-temperature hydrothermal methods have been employed to synthesise several ruthenate single crystals using KOH as mineraliser [36], and this could be extended to the iridate family.

While the pyrochlore iridates are a promising testbed for the theoretical predictions discussed here, some deviations from the ideal $J = 1/2$ Heisenberg model should be noted. Firstly, the Ir^{4+} ions in $RE_2Ir_2O_7$ are known to be Ising-like, with the spins preferring to point along the local [111] directions, i.e., into or out of the tetrahedra. It is this anisotropy, coupled with the AFM interactions, that gives rise to the AIAO ordered state at low temperatures and any reduction in the Ising-like nature of the spins (e.g., by substitution of a RE ion with a smaller radius) leads to a suppression of the transition temperature. Secondly, the Ir ions in these materials also exhibit a degree of mixing of the $J_{\text{eff}} = 1/2$ state with the $J_{\text{eff}} = 3/2$ states due to the local trigonal crystal field (although this is found to be minimised by replacement of the RE ion with some other species). The effects of these deviations from the ideal model are yet to be established, but these materials remain a promising avenue for exploration in this context.

---

[1] We also find that equal amplitude superpositions of dimer-singlet states vanish for small triangular lattices with periodic boundary conditions, but not for square, honeycomb and kagome lattices. We note that proofs of linear independence exist for dimer-singlet states on some of these lattices [37]; however, such proofs rely on open and not periodic boundary conditions.
[2] Y. Nagaoka, Phys. Rev. **147**, 392 (1966).
[3] D. J. Thouless, Proceedings of the Physical Society **86**, 893 (1965).

[4] S. Liang and H. Pang, Europhysics Letters **32**, 173 (1995).
[5] H. Park, K. Haule, C. A. Marianetti, and G. Kotliar, Phys. Rev. B **77**, 035107 (2008).
[6] G. Carleo, S. Moroni, F. Becca, and S. Baroni, Phys. Rev. B **83**, 060411 (2011).
[7] L. Liu, H. Yao, E. Berg, S. R. White, and S. A. Kivelson, Phys. Rev. Lett. **108**, 126406 (2012).
[8] E. Eisenberg, R. Berkovits, D. A. Huse, and B. L. Altshuler, Phys. Rev. B **65**, 134437 (2002).
[9] J. O. Haerter and B. S. Shastry, Phys. Rev. Lett. **95**, 087202 (2005).
[10] D. Poilblanc, Phys. Rev. Lett. **93**, 197204 (2004).
[11] C. N. Sposetti, B. Bravo, A. E. Trumper, C. J. Gazza, and L. O. Manuel, Phys. Rev. Lett. **112**, 187204 (2014).
[12] F. T. Lisandrini, B. Bravo, A. E. Trumper, L. O. Manuel, and C. J. Gazza, Phys. Rev. B **95**, 195103 (2017).
[13] S.-S. Zhang, W. Zhu, and C. D. Batista, Phys. Rev. B **97**, 140507 (2018).
[14] I. Morera, A. Bohrdt, W. W. Ho, and E. Demler, "Attraction from frustration in ladder systems," (2021).
[15] K.-S. Kim, Phys. Rev. B **107**, L140401 (2023).
[16] J. Hauschild and F. Pollmann, SciPost Phys. Lect. Notes , 5 (2018), code available from `https://github.com/tenpy/tenpy`.
[17] M. E. J. Newman and G. T. Barkema, *Monte Carlo Methods in Statistical Physics*, 1st ed. (Clarendon Press, 2011).
[18] We note however that evidence of possible nematic order has been seen in the quantum case in larger system sizes [38–40].
[19] M. Subramanian, G. Aravamudan, and G. Subba Rao, Progress in Solid State Chemistry **15**, 55 (1983).
[20] W. Witczak-Krempa and Y. B. Kim, Physical Review B **85**, 045124 (2012).
[21] K. Tomiyasu, K. Matsuhira, K. Iwasa, M. Watahiki, S. Takagi, M. Wakeshima, Y. Hinatsu, M. Yokoyama, K. Ohoyama, and K. Yamada, Journal of the Physical Society of Japan **81**, 034709 (2012).
[22] C. Donnerer, M. C. Rahn, M. M. Sala, J. G. Vale, D. Pincini, J. Strempfer, M. Krisch, D. Prabhakaran, A. T. Boothroyd, and D. F. McMorrow, Phys. Rev. Lett. **117**, 037201 (2016).
[23] H. Jacobsen, C. D. Dashwood, E. Lhotel, D. Khalyavin, P. Manuel, R. Stewart, D. Prabhakaran, D. F. McMorrow, and A. T. Boothroyd, Phys. Rev. B **101**, 104404 (2020).
[24] Y. Wang, T. F. Rosenbaum, D. Prabhakaran, A. T. Boothroyd, and Y. Feng, Physical Review B **101**, 220404(R) (2020).
[25] K. Matsuhira, M. Wakeshima, Y. Hinatsu, and S. Takagi, Journal of the Physical Society of Japan **80**, 094701 (2011).
[26] M. J. Coak, K. Götze, T. Northam De La Fuente, C. Castelnovo, J. P. Tidey, J. Singleton, A. T. Boothroyd, D. Prabhakaran, and P. A. Goddard, npj Quantum Materials **9**, 17 (2024).
[27] S. M. Disseler, C. Dhital, A. Amato, S. R. Giblin, C. de la Cruz, S. D. Wilson, and M. J. Graf, Phys. Rev. B **86**, 014428 (2012).
[28] H. Fukazawa and Y. Maeno, Journal of the Physical Society of Japan **71**, 2578 (2002).
[29] W. K. Zhu, M. Wang, B. Seradjeh, F. Yang, and S. X. Zhang, Phys. Rev. B **90**, 054419 (2014).
[30] J. J. Ishikawa, E. C. T. O'Farrell, and S. Nakatsuji, Phys. Rev. B **85**, 245109 (2012).
[31] D. Yanagishima and Y. Maeno, J. Phys. Soc. Jpn. **70**, 2880 (2001).
[32] H. S. Rajeev, P. Telang, and S. Singh, Solid State Communications **312**, 113863 (2020).
[33] H. Hoekstra and F. Gallagher, Inorg. Chem. **7**, 2553 (1968).
[34] J. N. Millican, R. T. Macaluso, S. Nakatsuji, Y. Machida, Y. Maeno, and J. Y. Chan, Materials Research Bulletin **42**, 928 (2007).
[35] K. Vlášková, P. Proschek, M. Diviš, D. Le, R. H. Colman, and M. Klicpera, Phys. Rev. B **102**, 054428 (2020).
[36] B. K. Patel, M. T. Kolambage, C. D. McMillen, and J. W. Kolis, Journal of Crystal Growth **602**, 126979 (2023).
[37] J. T. Chayes, L. Chayes, and S. A. Kivelson, Communications in Mathematical Physics **123**, 53 (1989).
[38] I. Hagymási, R. Schäfer, R. Moessner, and D. J. Luitz, Phys. Rev. Lett. **126**, 117204 (2021).
[39] N. Astrakhantsev, T. Westerhout, A. Tiwari, K. Choo, A. Chen, M. H. Fischer, G. Carleo, and T. Neupert, Phys. Rev. X **11**, 041021 (2021).
[40] M. Hering, V. Noculak, F. Ferrari, Y. Iqbal, and J. Reuther, Phys. Rev. B **105**, 054426 (2022).
